# The Legal Relations of Hospital, Hospital Staff, and Patient

**Published:** 1910-10-08

**Authors:** 


### THE LEGAL RELATIONS OF HOSPITAL, HOSPITAL STAFF, AND PATIENT.

The student who writes a thesis on a legal subject for the purpose of obtaining the degree of "LL.M." usually selects a topic of interest to lawyers rather than the general public. A thesis recently written at Cambridge is an exception to the rule. Mr. Digby Cotes-Preedy, who was seeking this degree, wrote on "The Legal Relations of Hospital Authorities, their Staff and Patients," and his interesting essay has now been published in pamphlet form.* As the subject is of considerable interest to our readers, we make no apology for referring to it somewhat fully.

General Conclusions.

In so far as statutes and decided cases are concerned, it is remarkable how few there are which have any bearing upon the subject in hand. It would almost seem as if the Legislature, so far as it has ever thought of hospitals, had adopted the opinion of Montaigne when he said "it would be better for us to have no laws at all than to have them in the prodigious numbers we have." As the essay before us shows, however, the responsibility of the governors of a hospital for the negligence of those employed on its staff has recently been considered by the courts in several important cases. These cases are fully dealt with in the pamphlet.

Mr. Preedy has drawn the following general conclusions from his examination of the authorities:—

"It is submitted, as regards patients,

"1. That a hospital authority owes to them the duty of exercising due care and skill in the selection of the members of its staff.

"2. That neither the relationship of master and servant, nor that of principal and agent exists between a hospital authority and its medical and surgical staff; and

"3. That neither the relationship of master and servant, nor that of principal and agent exists between a hospital authority and its nursing and household staff whilst under the control and direction of a member of the medical or surgical staff."

Their Justification.

A perusal of the various judgments upon which these propositions are founded serves to show that they are fully justified. Upon their face they are eminently satisfactory to the hospital authorities. Were the governors of these great charitable institutions to be held liable for the acts or omissions of all members of their staff, it is difficult to see where the liability would end. The most eminent surgeon sometimes makes a mistake; the most distinguished physician may sometimes give injudicious counsel. If the principles of *respondeat superior* were to be applied so as to make the hospital liable in such cases, the courts would be full of actions for malpractice. A plaintiff would naturally seek to draw water from the deepest well. He would launch his action against the hospital itself. The fact that he had been treated free gratis and for nothing would not soften the heart of the litigant.

Two Illustrative Cases.

It is interesting to examine one or two of the cases upon which Mr. Preedy has based his concise exposition of the law. Strange to say, the earlier decisions hail from America, where the proposition (1) (*supra*) was first enunciated in 1876. In a case

* Sweet and Maxwell. Price 1s.

which was heard that year, the Massachusetts General Hospital was sued for injuries alleged to have been sustained through the neglect and unskilful surgical treatment of certain members of the staff. The Supreme Court refused to entertain the action. In the course of his judgment, Judge Devens said: "It might well be questioned whether any contract could be inferred between the plaintiff and the defendant corporation. It has offered to him freely those ministrations which, as the dispenser of public charity, it had been able to provide for his comfort, and he has accepted them. . . . If, however, any contract can be inferred from the relation of the parties, it can be only on the part of the corporation that it shall use due and reasonable care in the selection of its agents."

### A New Zealand Case.

Here, then, is the *fons et origo* of that excellent learning which has declared that hospital authorities are not bound to insure the patients who enter the walls against negligence on the part of the medical or surgical staff. Many other American cases were decided in the same way and for the same reason. In a New Zealand case, too, the District of Auckland Hospital were sued for the consequences of the alleged negligence of their Medical Superintendent, who was said to be their servant. It was admitted that the board of the hospital had exercised due care in his appointment. In giving judgment, Mr. Joshua Williams said that the liability of the board was the same as that of a private person doing the same thing, and then continued: "If a benevolent individual undertook this work at his own cost, what would be his liabilities? . . . So far as medical and surgical treatment is concerned, I am satisfied if he had appointed a duly qualified medical man or surgeon, whose competence he had no reason to doubt, and with whose action he would have no right to interfere until he had reason to doubt his competence, that he has fulfilled his duty. Nor . . . can the doctor or surgeon be considered to be in the position of a servant to render the employer liable for his negligence."

### The St. Bartholomew's Case.

Seeing that the American and New Zealand cases were duly followed and approved by our own courts, Mr. Preedy was fully justified in alluding to them. The leading English case is Hillyer *v.* Governors of St. Bartholomew's Hospital, [1909] 2 K.B. 820. There the plaintiff, a medical man, sought damages for injuries due to the negligence of the defendants at their hospital. It appeared that the plaintiff entered the hospital to be examined under an anæsthetic. The examination was conducted by a consulting surgeon, chosen by the plaintiff himself, attached to the institution. For the purpose of the examination the plaintiff was so placed upon an operating table that his arms were permitted to hang over its side. In consequence of his left arm coming into contact with a hot-water tin projecting from beneath the table, the inner upper part of the limb was burned. The inner upper part of the right arm was bruised by the surgeon or some other person pressing against it during the examination. As a result of these injuries there followed paralysis of both arms and traumatic neuritis, which had since prevented the plaintiff from exercising his profession. Mr. Justice Grantham, having refused to leave the question of negligence to a jury on the ground that the governors were not responsible for the negligence of the consulting surgeon or the staff, even if proved, the plaintiff went to the Court of Appeal, where the judgment of the lower court was upheld. Lord Justice Farwell, after approving the American decisions already referred to, said: "It is, in my opinion, impossible to contend that Mr. L., the surgeon, or the acting assistant surgeon, or the acting house surgeon, or the administrator of anæsthetics, or any of them, were servants in the proper sense of the word; they are all professional men, employed by the defendants to exercise their profession to the best of their abilities according to their own discretion; but in exercising it they are in no way under the orders or bound to obey the directions of the defendants."

### Nurses and Attendants.

So much for the medical men concerned. The real interest of this case lies in what was said by the Lords Justices as to the position of the nurses and the two carriers. With regard to them, Lord Justice Farwell said: "The three nurses and the two carriers stand on a somewhat different footing, and I will assume that they are the servants of the defendants. But although they are such servants for general purposes they are not so for the purposes of operations and examinations by the medical officers. If and so long as they are bound to obey the orders of the defendants, it may well be that they are their servants, but as soon as the door of the operating theatre or room has closed on them for the purposes of an operation (in which term I include examination by the surgeon) they are under the sole orders of the operating surgeon until the whole operation has been completely finished; the surgeon is for the time being supreme, and the defendants cannot interfere with or gainsay his orders. . . . The nurses and carriers, therefore, assisting at an operation cease for the time being to be the servants of the defendants, inasmuch as they take their orders during that period from the operating surgeon alone, and not from the hospital authorities."

It follows that where the facts are similar to those proved in Hillyer's case, the hospital authorities will escape liability on the ground that the nurses are, for the time, subject to the orders and directions of the doctors. It was upon this that Mr. Preedy founded his third proposition.

### Liability of Hospitals.

The same case, however, may be regarded as an authority for the statement that there are certain duties to be performed by nurses, for the negligent performance of which the governors may be held responsible. The point was put very clearly by

Lord Justice Kennedy, who said: "It may well be, and for my part I should, as at present advised, be prepared to say that the hospital authority is legally responsible to the patients for the due performance of their servants within the hospital of their purely ministerial or administrative duties, such as, for example, attendances of nurses in the wards, the summoning of medical aid in cases of emergency, the supply of proper food, and the like. The management of a hospital ought to make, and does make, its own regulations in such matters of routine, and it is, in my judgment, legally responsible to the patients for their sufficiency, their propriety, and observance of them by the servants." Lord Collins apparently held the same view, for towards the close of his judgment in another case (Hall and Wife *v.* Lees) he said: "The relation of master and servant did not exist between them (the nursing association) and the nurse for that work (attendance on the female plaintiff), though possibly for other purposes it might exist."

### The Question of Probationers.

As the author of the thesis points out, this question will probably demand attention before long, having regard to the fact that partially trained nurses are often employed in the hospitals. In an American case, it appeared that the plaintiff was injured owing to the negligence of a nurse who had only completed nine months of training, the full course being two years. The plaintiff having been non-suited when her action for damages first came on for hearing, the Supreme Court directed a new trial. Judge Barrett said: "It was for the jury to say whether, in furnishing this careless pupil of limited experience the defendant fulfilled its contract obligation to the plaintiff; and if it did not, and injury resulted from the breach of that obligation, to award her adequate compensation."

It frequently happens that an examination of the case law applicable to any subject suggests some defect which ought to be amended by legislation. It must be confessed that, in the present instance, no amendment can be suggested. It is clear law that the physician or surgeon who treats a patient gratuitously is liable for negligence. The law is clear, even if the doctrine is harsh. But the practitioner is not bound to minister to all comers. Within certain limits the hospital, which is generally a charitable institution, must open its gates to the public. The money of pious founders and generous donors is not intended for the payment of damages to persons who allege that they have been improperly treated.

